# High-Density Morphometric Analysis of Shape and Integration: The Good, the Bad, and the Not-Really-a-Problem

**DOI:** 10.1093/icb/icz120

**Published:** 2019-06-27

**Authors:** Anjali Goswami, Akinobu Watanabe, Ryan N Felice, Carla Bardua, Anne-Claire Fabre, P David Polly

**Affiliations:** 1 Life Sciences Department, Vertebrates Division, Natural History Museum, London, SW7 5BD, UK; 2 Department of Genetics, Evolution and Environment, University College London, London, WC1E 6BT, UK; 3 Department of Anatomy, New York Institute of Technology College of Osteopathic Medicine, Old Westbury, NY 11568, USA; 4 Division of Paleontology, American Museum of Natural History, New York, NY 10024, USA; 5 Department of Cell and Developmental Biology, Centre for Integrative Anatomy, University College London, London, WC1E 6BT, UK; 6 Departments of Earth and Atmospheric Sciences, Biology, and Anthropology, Indiana University, 1001 E. 10^th^ Street, Bloomington, IN 47405, USA

## Abstract

The field of comparative morphology has entered a new phase with the rapid generation of high-resolution three-dimensional (3D) data. With freely available 3D data of thousands of species, methods for quantifying morphology that harness this rich phenotypic information are quickly emerging. Among these techniques, high-density geometric morphometric approaches provide a powerful and versatile framework to robustly characterize shape and phenotypic integration, the covariances among morphological traits. These methods are particularly useful for analyses of complex structures and across disparate taxa, which may share few landmarks of unambiguous homology. However, high-density geometric morphometrics also brings challenges, for example, with statistical, but not biological, covariances imposed by placement and sliding of semilandmarks and registration methods such as Procrustes superimposition. Here, we present simulations and case studies of high-density datasets for squamates, birds, and caecilians that exemplify the promise and challenges of high-dimensional analyses of phenotypic integration and modularity. We assess: (1) the relative merits of “big” high-density geometric morphometrics data over traditional shape data; (2) the impact of Procrustes superimposition on analyses of integration and modularity; and (3) differences in patterns of integration between analyses using high-density geometric morphometrics and those using discrete landmarks. We demonstrate that for many skull regions, 20–30 landmarks and/or semilandmarks are needed to accurately characterize their shape variation, and landmark-only analyses do a particularly poor job of capturing shape variation in vault and rostrum bones. Procrustes superimposition can mask modularity, especially when landmarks covary in parallel directions, but this effect decreases with more biologically complex covariance patterns. The directional effect of landmark variation on the position of the centroid affects recovery of covariance patterns more than landmark number does. Landmark-only and landmark-plus-sliding-semilandmark analyses of integration are generally congruent in overall pattern of integration, but landmark-only analyses tend to show higher integration between adjacent bones, especially when landmarks placed on the sutures between bones introduces a boundary bias. Allometry may be a stronger influence on patterns of integration in landmark-only analyses, which show stronger integration prior to removal of allometric effects compared to analyses including semilandmarks. High-density geometric morphometrics has its challenges and drawbacks, but our analyses of simulated and empirical datasets demonstrate that these potential issues are unlikely to obscure genuine biological signal. Rather, high-density geometric morphometric data exceed traditional landmark-based methods in characterization of morphology and allow more nuanced comparisons across disparate taxa. Combined with the rapid increases in 3D data availability, high-density morphometric approaches have immense potential to propel a new class of studies of comparative morphology and phenotypic integration.

## Introduction

**Fig. 1:**
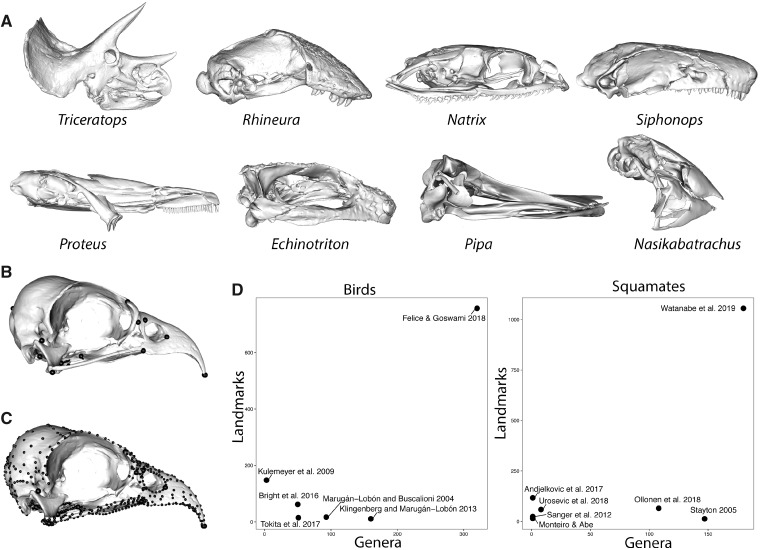
Characterization of morphologically disparate taxa. (**A**) The disparity of biological shapes and presence and absence of homologous structures, as exemplified in the skulls of diapsids and amphibians and (**B**) the difficulty of locating discrete landmarks in some taxa, such as the strongly sutured skulls of birds, present challenges for the quantitative analysis of morphology. High-density semilandmarks (**C**) can capture the morphology of complex regions with far more detail and allow for comparisons of homologous structures across disparate taxa, resulting in (**D**) massive increases in dataset size for studies of comparative morphology. Bird data in (**B**, **C**) from Felice and Goswami (2018).

Big data approaches to morphological studies have entered a new phase in recent years, due to the ubiquity of high-resolution imaging tools, such as micro-computed tomography imaging and surface scanning and photogrammetry (Davies et al. 2017). Open databases (Morphosource, Phenome10K, Digimorph, Morphomuseum, and institutional sites) now host three-dimensional (3D) image files for tens of thousands of specimens, meaning that obtaining access to 3D scans representing a substantial proportion of the extant, and even extinct diversity, for clades as large as all vertebrates, is rapidly become the expectation, rather than a pipe dream. These new datasets open new possibilities for investigating biological questions (Collyer et al. 2015), including comparative analyses that can begin to quantify and analyse morphology at an extremely high level of detail across wider taxonomic scales (Fig. 1).

To date, most comparative studies using geometric morphometrics comparing morphology in a quantitative framework have either sampled closely related taxa that share substantial numbers of landmarks of unambiguous homology (i.e., Type I/II landmarks following Bookstein (1991)) or sample a broader taxonomic scope but by using a much reduced number of landmarks. Alternatively, analyses may use traditional metrics, such as linear measurements, which capture some aspect of the morphology of functionally analogous regions (e.g., rostrum) that can be compared directly across diverse taxa, but provide very limited detail on morphology and cannot be used to reconstruct shape (Marugán-Lobón and Buscalioni 2003). Recent years have seen development and refinement of geometric morphometric expansions of alternatives to homologous landmarks (Bookstein 1991), with application of 3D sliding semilandmarks or pseudolandmarks. Published definitions of semilandmarks and pseudolandmarks are inconsistent and often interchangeable, but here, we refer to semilandmarks as those whose initial position is relative to landmarks with biological homology, whereas pseudolandmarks are entirely automatically placed without reference to anatomically defined landmarks, for example, by sampling uniformly from a surface mesh (e.g., auto3dgm, Boyer et al. 2015; Generalized Procrustes Surface Analysis, Pomidor et al. 2016). Detailed descriptions, discussions, and comparisons of these methods (Adams et al. 2004, 2013; Bardua et al. 2019a; Bookstein et al. 2002; Boyer et al. 2015; Gonzales et al. 2016; Gunz and Mitteroecker 2013; Gunz et al. 2005; Mitteroecker and Gunz 2009; Rohlf and Marcus 1993; Vitek et al. 2017; Zelditch et al. 2004) demonstrate the promise these methods offer for quantifying regions that are poorly characterized by use of only discrete landmarks, due to the lack of unambiguous homology across specimens or the presence of large areas without any appropriate structures at which to place landmarks. The lack of points of unambiguous homology becomes increasingly challenging with comparative studies across large clades. For example, ongoing work by our research team on tetrapod skulls identified a total of 12 Type I landmarks that could be reliably placed across the full cranial diversity of that clade, meaning that the vast majority of cranial morphology would go unsampled (Fig. 1). Even for less speciose clades, such as the 32 extant genera of caecilian amphibians, this can be a highly limiting factor due to a large degree of variation in bone presence and suture patterns (Bardua et al. 2019b). The second point is an issue at any scale of analysis, as many structures will only have discrete points, such as sutures, at their boundaries, meaning that most of the shape of the structure will be unsampled. For example, even in a clade with relatively conserved morphology such as birds, a high degree of bone fusion has limited previous studies to a small number of landmarks (e.g., 11–17 landmarks in Bright et al. [2016]; Klingenberg and Marugán-Lobón 2013) (Fig. 1).

While semilandmarks and pseudolandmarks are now frequently deployed to circumvent these landmark-only issues (Polly 2008), questions have been raised about their necessity and applicability for the study of phenotypic integration and other topics in which the covariance structure of shape data is important (Cardini 2019; Lele and Richtsmeier 1990; Richtsmeier and Lele 2001). Phenotypic integration refers to the correlation or covariance of traits due to genetic, developmental, or functional interactions (Olson and Miller 1958), and analysis of these relationships among traits relies on accurate quantification of their morphology and their correlations or covariances. Pseudolandmarks have not yet been used in studies of integration, and their use in such studies is likely hindered by their lack of reference to biological homology. In contrast, many studies have used semilandmarks to quantify the relationships among different elements or regions of structures ranging from the vertebrate skulls and mandibles (e.g., Bardua et al. 2019a, 2019b; Felice and Goswami 2018; Marshall et al. 2019; Parr et al. 2016; Watanabe et al. 2019; Zelditch et al. 2009) to fish fins (Larouche et al. 2018; Du et al. 2019) to trilobite cranidia (Webster and Zelditch 2011). For this reason, we focus here on the use of semilandmarks (and more specifically, sliding semilandmarks) in studies of phenotypic integration, and more broadly, on their contribution to comparative studies of morphological evolution.

The concerns about using semilandmarks for such analyses fall into two categories. First, and most broadly, all geometric morphometric data, including Type I/II landmarks as well as semilandmarks, require registration prior to analysis in order to remove the non-shape aspects of position, orientation, and isometric size. The most common method of registering specimens is generalized Procrustes superimposition (Rohlf 1990; Rohlf and Slice 1990), which is a least-squares approach that minimizes variance across an entire landmark (and/or semilandmark) configuration and rescales each configuration to unit centroid size. Because this approach minimizes variance across the entire configuration, it can have the effect of spreading variance across landmarks. In other words, it may shift variance from more variable landmarks to less variable ones and imposes a common scaling on a structure that may have differential scaling in different regions (Baab 2013; Klingenberg 2009), both of which can alter the covariance structure of the landmarks and change the inferred pattern of integration among traits. It has been recently asserted that this effect may be exacerbated in larger geometric morphometric datasets, such as those generated through the application of semilandmarks, although such an effect was not demonstrated, and assumed that the effects would reduce the ability to detect biological modularity in data (Cardini 2019). Second, and more specifically, it has also been asserted that closely packed semilandmarks may falsely inflate the pattern of modularity (the division of structures into highly-integrated, but semi-independent subunits) because the position of each semilandmark is conditional on its neighbors and therefore multiplication of semilandmarks could increase the total covariance within a putative module. For these reasons, it has been suggested that “big data” is not necessarily better data when it comes to geometric morphometric analyses, especially analyses of phenotypic integration and modularity (Cardini 2019).

Here, we examine these issues and their potential impact on phenomic analyses of phenotypic integration. To do so, we first assess whether the gains are worth these potential drawbacks by considering: (1) do high-density semilandmark datasets actually capture shape better than Type I/II landmark data? If so, we then consider the practical consequences of using these high-density data, or geometric morphometric data more generally, for analyses of phenotypic integration, by addressing: (2) does Procrustes superimposition mislead analyses of phenotypic integration and modularity; and (3) how do analyses of integration with high-dimensional semilandmarks compare to those with only landmarks?

## The effect of high-density geometric morphometric data on shape analyses

**Fig. 2:**
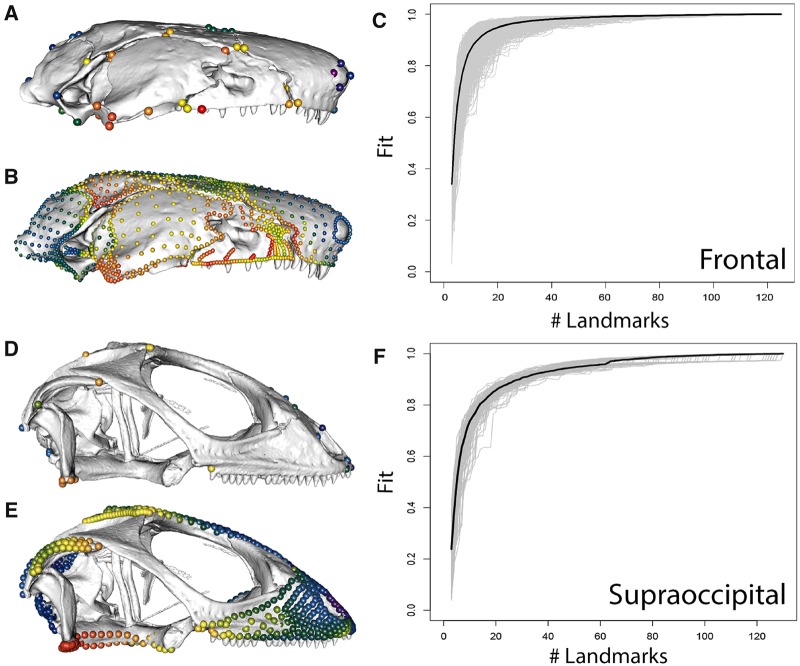
Landmark-only (**A, D**) and full landmark and semilandmark configurations (**B, E**) for caecilians (**A, B**) and squamates (**D, E**), and landmark sampling curves generated by LaSEC for (**C**) the frontal bone of caecilians and (**F**) the supraoccipital of squamates. Colours in **A, B, D,** and **E** indicate Procrustes variance at each landmark position, demonstrating that full and landmark-only configurations produce similar overall patterns but that some areas of high or low variance are entirely unsampled in landmark-only analyses. Sampling curve (**C, F**) illustrate that 25–35 landmarks and semilandmarks are required to confidently and robustly characterize the shape variation in these individual bones. Caecilian data from Bardua et al. (2019b), and squamate data from Watanabe et al. (2019).

To quantify whether high-density semilandmark data add important additional information on morphology, we analysed two datasets. The first dataset is from a recently published study of the cranium of caecilian amphibians (Fig. 2A, B), with 16 cranial regions quantified across 32 genera using 53 landmarks and 687 curve and 729 surface sliding semilandmarks (Bardua et al. 2019b). The second is a recently published dataset of squamates (Fig. 2D, E), with 13 cranial regions quantified in 174 species with 47 landmarks and 595 curve and 580 surface sliding semilandmarks (Watanabe et al*.* 2019). To examine how many landmarks/semilandmarks are required to capture the shape of a region in these datasets, we implemented Landmark Sampling Evaluation Curve (LaSEC) analysis, using the ‘lasec’ function in the R package LaMDBA (Watanabe 2018). This function subsamples the original dataset through random addition of landmarks and semilandmarks, determining the fit of each reduced dataset to the complete dataset, and repeating this for a selected number of iterations. Fit is calculated based on Procrustes distance between the full and subsampled datasets with respect to position of the specimens in high-dimensional morphospace (i.e., not position of the landmarks). We performed LaSEC for (1) landmarks-only and (2) subsampled landmarks and semilandmarks (curve and surface points) for the caecilian and squamate datasets, for individual cranial regions. The function generates a sampling curve (Fig. 2C, F), where a plateau in the curve signifies stationarity in characterization of shape variation and fewer landmarks than the plateau indicates inadequate characterization. We compared the fit of the landmark-only and full datasets and also determined the number of landmarks and semilandmarks that would have been sufficient for each region, given a required fit of 0.9, 0.95, and 0.99 between the reduced and complete datasets (Tables 1 and 2). To compare the relative contribution of curve and surface semilandmarks to shape characterization, we further conducted LaSEC analysis comparing the fit of landmarks and curve sliding semilandmarks to the full dataset of landmarks and curve and surface sliding semilandmarks to the squamate dataset.

**Table 1:** Results from performing LaSEC with 1000 iterations on individual cranial partitions of extant caecilian datasets

Structure	# landmarks	# landmarks + semilandmarks	Fit = 0.90	Fit = 0.95	Fit = 0.99	Fit of landmark-only dataset
Basisphenoid	4	155	15	25	69	0.583
Frontal	4	125	13	21	61	0.617
Jaw joint	3	50	13	19	37	0.306
Maxillopalatine (interdental shelf)	4	110	13	19	52	0.782
Maxillopalatine (lateral surface)	3	134	14	23	64	0.238
Maxillopalatine (palatal surface)	5	75	13	19	44	0.602
Nasopremaxilla (dorsal surface)	7	148	13	21	61	0.684
Nasopremaxilla (palatal surface)	3	59	8	12	29	0.770
Occipital condyle	2	34	11	15	27	NA (only two landmarks)
Occipital region	5	153	16	27	73	0.605
Parietal	3	126	11	18	51	0.361
Pterygoid	–	50	7	10	24	NA
Quadrate (lateral surface)	2	57	12	18	38	NA (only two landmarks)
Squamosal	4	104	15	25	61	0.574
Stapes	–	20	10	12	17	NA
Vomer	3	69	12	18	41	0.538

Values for Fit = 0.9, 0.95, and 0.99 denote the median number of randomly subsampled landmarks degree of fit (0 to 1) of randomly subsampled landmark configurations and fixed-only datasets to the respective full high-dimensional coordinate data. Separate analysis of landmarks + curve sliding semilandmarks was not conducted for caecilians, as curves for some regions (e.g., maxillopalatine) were not homologous and removed prior to analyses. For details and definitions of cranial regions, see Bardua et al. (2019b).

**Table 2:** Results from performing LaSEC with 1000 iterations on individual cranial partitions of extant squamate datasets

Structure	# LMs	# curve sLMs	# surface sLMs	Fit = 0.90	Fit = 0.95	Fit = 0.99	Fit of landmark-only dataset	Fit of landmark + curve dataset
Premaxilla	4	35	39	15	23	49	0.713	0.981
Nasal	4	40	42	15	25	54	0.664	0.977
Maxilla	5	65	92	16	27	74	0.696	0.913
Jugal	3	60	31	13	20	51	0.645	0.962
Frontal	4	40	86	14	25	66	0.721	0.993
Parietal	4	60	34	16	28	64	0.647	0.987
Squamosal	3	30	19	17	25	43	0.452	0.993
Jaw joint	4	20	18	20	27	38	0.484	0.999
Supraoccipital	5	60	67	30	55	90	0.597	0.979
Occipital condyle	–	15	22	22	27	34	N/A	0.988
Basioccipital	4	60	58	14	26	66	0.805	0.982

Values for Fit = 0.9, 0.95, and 0.99 denote the median number of randomly subsampled landmarks required for respective degree of fit of randomly subsampled landmark configurations to the respective full (landmark + curve and surface sliding semilandmark) dataset. Fit of landmark-only and landmark + curve sliding semilandmark datasets compared to full dataset is also provided for comparison, demonstrating that the addition of curve sliding semilandmarks alone greatly improves representation of shape over landmark-only analyses (although see discussion regarding issues with curves for some highly-variable structures in the caecilian skull). The occipital condyle, pterygoid, and palatine are not listed as they lack either unique landmarks or surface sliding semilandmarks for some taxa. For details definitions of cranial regions, see Watanabe et al. (2019).

These analyses demonstrate that landmark-only datasets do not fully capture the variation of these analysed structures, with the fit between landmark-only and full landmark plus semilandmark datasets ranging between 0.24 and 0.81 for individual cranial regions. To achieve a fit of 0.95 to a high-density dataset, cranial regions need to be sampled by >20 landmarks and semilandmarks. While this cannot distinguish between the value of large numbers of landmarks and similarly large numbers of curve and/or surface sliding semilandmarks, it is uncontroversial that semilandmarks can sample more morphology than Type I/II landmarks. In these datasets, for example, our attempt to maximize representation of cranial structures with Type I/II landmarks resulted in 2–7 landmarks sampled per region, in comparison to the >20 landmarks and semilandmarks that our analyses estimated, which are needed to represent the variation in each region. Thus, landmark data alone are insufficient to fully characterize morphological variation for many datasets. In terms of the respective contribution of curve and surface sliding semilandmarks to characterizing variation, the addition of curve sliding semilandmarks alone is a vast improvement on landmark-only analyses, with a fit of >0.9 for all cranial regions in squamates and approaching a near perfect fit to the full dataset for relatively flat structures. However, it is important to note that the reason a similar analysis would be less informative, and thus was not conducted, for the caecilian dataset, is that some of the most variable regions, including the maxillopalatine and pteryoid, required the use of some non-homologous curves to accommodate variably present structures, such as the tentacular canal (Bardua et al. 2019a, 2019b). These curves were then excluded, with only landmarks and surface sliding semilandmarks used in further analyses. Thus, although curves may capture much of the morphological variation of the full landmark, curve, and surface dataset for many structures, they can be problematic and inapplicable in some of the most interesting, highly variable regions, particularly as comparisons expand across increasingly disparate taxa. Similarly, surface points cannot always be applied to all structures, such as the extremely narrow palatal region of snakes. Both curve and surface sliding semilandmarks provide important and complementary information on shape variation and our results demonstrate that both are improvements over analyses of landmarks alone for characterizing complex morphologies.

This result is further demonstrated by examining patterns of variance across landmarks and semilandmarks (Fig. 2). While the overall distribution of variance is similar in both datasets, large areas of the cranium are unsampled in landmark-only datasets, and thus some regions that are highly variable across taxa, such as the maxillopalatine of caecilians, are inadequately represented by landmarks. Thus, high-density configurations clearly contain important aspects of shape variation that is not captured by landmark-only analyses.

## The effect of Procrustes superimposition on analyses of modularity

In order to assess how Procrustes superimposition impacts covariance patterns between landmarks and the ability to recover modular patterns from them, we performed a controlled series of simulation experiments in which we varied the degree of variability at each landmark, the direction of covariation, and the number of landmarks. Each experiment is described in detail below.

**Fig. 3:**
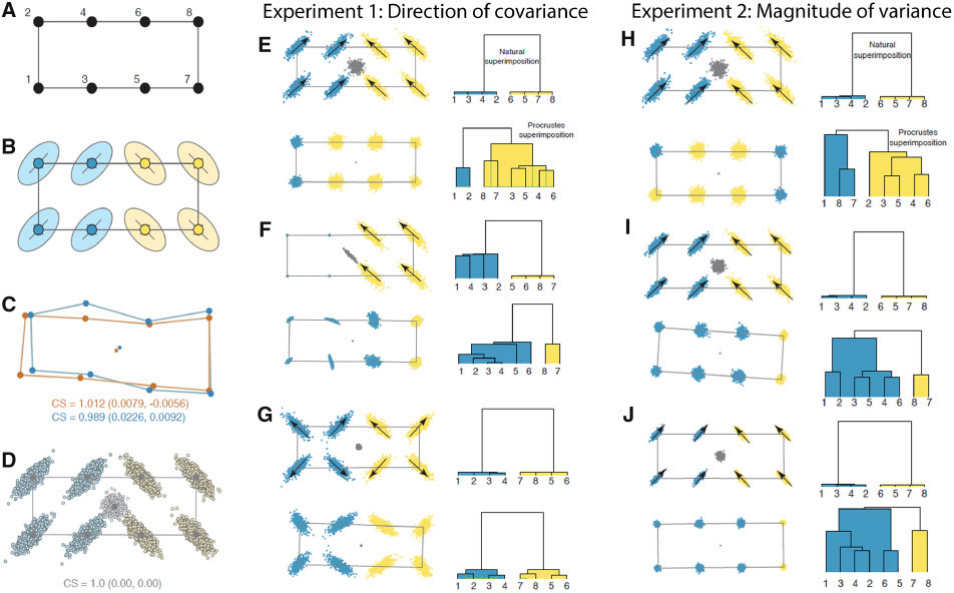
Simulation experiments 1 and 2 of the effect of Procrustes superimposition on covariance patterns and recovery of biological modules. Starting with a base archetype (**A**), we perturbed variances and covariances (**B**) in each experiment, with resultant effects on shape centroids (**C**), to generate a sample of “naturally superimposed” shapes (**D**), which are then subjected to Procrustes superimposition. In Experiment 1, we test the effect of direction of covariance, with covariances of two modules set at 90° to each other (**E**), one module of invariant landmarks (**F**), and both modules with covariances oriented away from their respective centroids (**G**). In Experiment 2, we vary the magnitude of variance, with variances initially identical to that of Experiment 1 (**H**), and then reduced to 80% (**I**) and 60% (**J**). For each experiment, landmark configurations are shown on the left, and clusters of recovered modules are shown on the right.

Experimental samples were modeled by randomly perturbing landmarks around a base configuration (or “*archetype”*; Fig. 3A) based on a multivariate normal covariance matrix **V** that we varied systematically with each experiment (Fig. 3B). Each instance of **V** was given two modules in which covariances among landmarks (and semilandmarks) within modules was higher than between modules. The number of rows and columns (landmark coordinates) in **V** and the magnitude of their covariances was varied to match the conditions of each experiment. Residual variation was then simulated by post-multiplying the Cholesky decomposition of **V** by a *kp* × *n* matrix of points drawn from *n* univariate normal distributions with mean of 0 and variance *v*, where *k* is the number of landmarks (and semilandmarks), *p* is the dimensionality of each landmark (or semilandmark), and *n* is the number of individuals in the sample. This multiplication produces a matrix of *n* individuals with *kp* landmarks (and semilandmarks) with covariance **V**. Finally, the residuals were added to the base configuration of landmarks (and semilandmarks) to produce a sample of shapes (Fig. 3D). Each simulated dataset consisted of 500 individual shapes unless otherwise noted.

Note that covariance between the *x* and *y* (and *z*) axes of a landmark produces a scatter of variation that has a directional orientation. For example, if a landmark has equal variances in both the *x* and *y* axes, any covariance between them will produce an ellipse of points with a major axis at an angle of 45°. For convenience, all coordinates were given the same variance, which produced this 45° angle in all landmarks (either in a positive or negative direction). For experiments where a more directionally complex covariance pattern was desired, individual scatters of simulated residual points were rotated into new orientations (i.e., the ellipsoids in Fig. 3B were pivoted around their corresponding landmark into new orientations), which is equivalent to altering the variances and covariances of their coordinates.

In each experiment, we assessed the effect of Procrustes superimposition on recoverability of modules using two metrics: (1) we tested whether the original modular pattern was significantly supported after Procrustes superimposition using the covariance ratio (CR) coefficient randomization test (Adams 2016) and (2) we compared the modules recovered from the original and Procrustes superimposed shapes using hierarchical clustering analysis. The CR test determines whether the ratio of covariation within and between the original modules is strongly enough preserved to produce a statistically significant correlation compared to randomized modules. CR values are high when between module correlations are higher than within module correlations (i.e., when modules are not distinct) and they decline toward 0 as modularity becomes stronger. Significance is tested by randomizing landmarks between modules and comparing the observed CR value with the distribution of randomized values (Adams 2016). The hierarchical clustering analysis used Ward’s minimum variance linkage algorithm on a *k × k* covariance matrix using canonical correlations between landmarks (Goswami and Polly 2010). This approach minimizes total within-cluster variance to cluster landmarks and was used to determine whether the same organization of traits (i.e., modules) was recovered before and after Procrustes superimposition and whether that pattern matched the modules constructed in **V**. Hereafter, we refer to the original simulated shapes before Procrustes superimposition as “naturally superimposed,” and we discuss the assumptions and implications of that concept further below. The number of significant modules in each cluster was estimated by comparing the observed eigenvalue structure to a null distribution derived from a Monte Carlo simulation using the same base shape but with zero covariance with 100 iterations (see Goswami and Polly 2010; Polly and Goswami 2010). All analyses were performed in *Mathematica* (Wolfram Research, 2018) using the *Modularity for Mathematica* (v. 2.0) and *Geometric Morphometrics for Mathematica* packages (Polly 2019; Polly and Goswami 2010).

### Experiment 1: Direction of covariance

In this experiment, the direction of landmark covariance was systematically altered (Fig. 3E–G). A simple *archetype* of eight landmarks arranged in a rectangle with two modules of four landmarks symmetrically arranged to the left and right of the *archetype’*s centroid was used. Correlations between landmarks within each module was set at 0.8, except for the second test where one module was given completely invariant landmarks except for a small amount of uncorrelated noise. In the first test, the orientation of covariance in the left module was set at positive 45° with respect to the length of the archetypal rectangle and in the right module it was set at 135°, which is 90° to the first module (Fig. 3E). In the second test, the left module had four invariant landmarks and the right module was identical to the right module in the first test (Fig. 3F). In the third test of this experiment, the orientation of variation in both modules was such that each landmark had a positive covariance pointing away from its respective module’s center (Fig. 3G).

In the first test in this experiment, Procrustes superimposition altered the covariance pattern so much that the original modules were unrecoverable. Despite having a strongly modular pattern that was easily recovered from the naturally superimposed data, the modules were not recovered from the Procrustes superimposed shapes. The pattern of covariance was strongly altered by Procrustes superimposition, which is seen visually in Fig. 3E and indicated by their comparatively high CR value (CR = 1.27; *P* = 0.94). Note that the centroids of the original shapes are highly variable in their position, with an unconstrained scatter that is nearly as large as the scatter of points around any of the landmarks (Fig. 3E). The stability of the centroid point turns out to be an important factor determining how much Procrustes superimposition alters the covariance pattern of the landmarks.

The second test, in which one module consisted of invariant landmarks, performed no better and arguably worse in terms of module recoverability (Fig. 3F). The two modules were not recoverable even from the naturally superimposed data, largely because the “invariant” module is not truly modular because its landmarks do not covary. The dendrogram based on the naturally superimposed shapes recovered a tight cluster between the four landmarks in the right module, but they were not significantly distinguished from the landmarks of the left “module” based on the eigenvalue variance randomization tests. Similarly, only one module was recovered from the Procrustes superimposed data, but there was no hint of similarity between the landmarks of the right module in the dendrogram. CR was also high and non-significant (CR = 1.14; *P* = 0.30). The position of the centroid of the naturally superimposed shapes was more constrained than in the first test, although it was still quite variable.

In the third test, in which the direction of variation was symmetrically radial in each module instead of perfectly parallel, the true modular pattern was easily recovered (Fig. 3G). Variability in the position of the centroid in the naturally superimposed shapes was much less than in the previous two tests, and much smaller than the variability at individual landmarks. The relative consistency of the position of the centroid is a result of the symmetry of the landmark variability. Because the original centroids are close together, changes in the overall pattern of covariance due to Procrustes superimposition are small. The CR test indicated that the original modules were recoverable after Procrustes superimposition (CR = 0.51; *P* < 0.001).

This experiment suggests that the symmetry (or lack thereof) in the directions of covariance patterns within and between modules affects variability in position of the centroid from one shape to the next and that the degree of variation in the position of the centroid relative to variation in individual landmarks is a major determinant of how much Procrustes superimposition, which recenters shapes on their centroids, alters the covariance structure.

### Experiment 2: Magnitude of variance

One possible interpretation of the first experiment is that the less variation there is in shape, the more constrained will be the position of the centroid and the less the covariance pattern will be altered by Procrustes superimposition. In the second experiment, we therefore tested whether the magnitude of shape variation has an effect on recoverability of modular patterns; it does not.

This experiment used the same directional covariance structure as in the first test of the previous experiment (Fig. 3E) but systematically varied the amount of variance in the landmark coordinates (Fig. 3H–J). The first test in Experiment 2 was stochastically identical to the first test in Experiment 1 (CR = 1.25; *P* = 0.93). In the second and third tests, the variance at each landmark was reduced to 80 and 60%, respectively (and the strength of covariance was maintained at 0.8). Even though variation in the position of the centroid was progressively smaller in the second and third tests (Fig. 3I, J), the CR coefficient remained approximately the same (CR = 1.24 and 1.25; *P* = 0.90 and 0.93) and the original modules were not recovered from the Procrustes superimposed data.

Even though the centroid position was less variable in the second and third tests, the effect of Procrustes superimposition on the covariance structure remained approximately constant because the centroid remained just as variable with respect to the variation at the individual landmarks. The translational and rotational components of Procrustes superimposition therefore had a proportionally similar effect on the relative positions of the landmarks (and therefore their covariance structure) regardless of the absolute magnitude of shape variation. This experiment shows that it is not the magnitude of shape variation *per se* that matters.

### Experiment 3: Number of landmarks

**Fig. 4:**
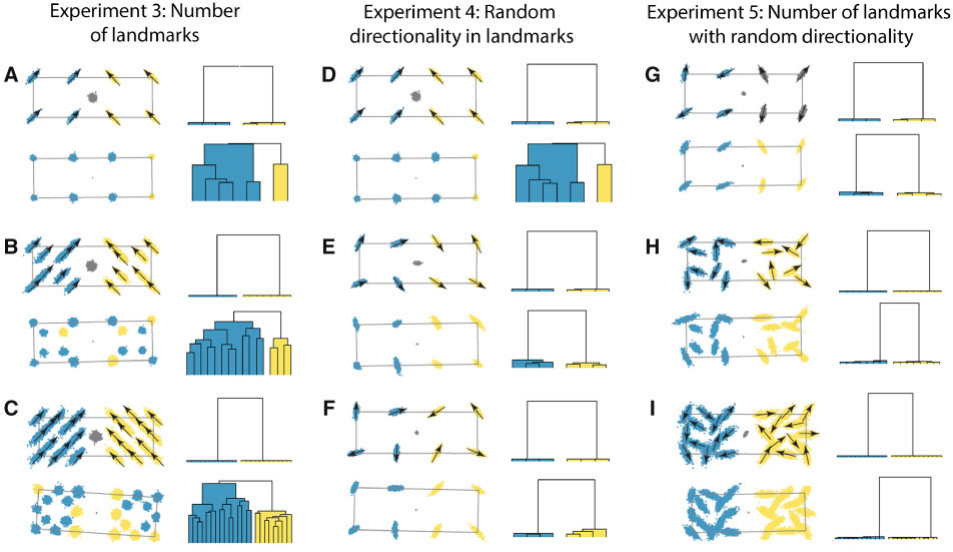
Simulation experiments 3–5 of the effect of Procrustes superimposition on covariance patterns and recovery of biological modules. In Experiment 3, we increase landmark numbers from the 8 landmarks of Experiment 1 (**A**), to 16 landmarks (**B**), and 24 landmarks (**C**). In Experiment 4, we vary the directionality of landmarks, from the symmetric variation of Experiment 1 (**D**) to random directions of variation (**E, F**). Finally, in Experiment 5, we combine the effects of Experiments 3 and 4, by randomly rotating landmarks for the initial set of 8 landmarks (**G**), and then 16 landmarks (**H**) and 24 landmarks (**I**). For each experiment, landmark configurations are shown on the left, and clusters of recovered modules are shown on the right.

The third experiment doubled and tripled the original number of landmarks to determine whether additional landmarks help minimize the effect of Procrustes superimposition (Fig. 4A–C). They do not (at least not without the contribution of other factors, as explained below). The first test in this experiment (Fig. 4A) was stochastically identical to that in Fig. 3E (CR = 1.28; *P* = 0.96). In the second test, four new landmarks were added to each module positioned one-quarter of the way toward the respective center of the module (Fig. 4B). In the third test, four more landmarks were added, these equidistant from the original four landmarks along the periphery of each module (Fig. 4C). The direction of covariation of the new landmarks in each module was identical to its original four.

The addition of landmarks had no substantial effect on variation in the position of the centroid of the naturally superimposed shapes, and only minor improvements in the CR test (CR = 1.11 and 1.09; *P* = 0.88 and 1.00) and offered no improvement in the recoverability of modules. Because the additional landmarks covary in the same direction and with the same magnitude as the original landmarks, they do not constrain the position of the centroid and are thus equally affected by the Procrustes superimposition process. Therefore, the effects of Procrustes superimposition on covariance structure are not increased by the addition of landmarks (or semilandmarks), *contra*Cardini (2019), but neither are they decreased.

### Experiment 4: Direction of covariance II

The first three experiments indicate that Procrustes superimposition has a strong effect on the covariance matrix, and thus recoverability of modules, when variation in position of the centroid is only loosely constrained relative to variation in the individual landmarks. Neither the absolute variability nor the number of the landmarks has an effect, but the overall pattern of directionality of covariation in the landmarks does. The effect of Procrustes superimposition was minimized in the third test of the first experiment when directionality of variation was symmetric with respect to both the center of each module and the centroid of the entire shape.

Next, we tested how random patterns of directional variation within and between modules affect recoverability of modules (Fig. 4D–F). Variation in real biological structures is much more directionally complex than any of the examples tested in the first experiment (e.g., Zelditch et al. 1993). It is difficult to imagine a biological example in which trait variation across a complex morphology is structured in entirely parallel or perpendicular directions. Thus, in this experiment we randomly oriented the direction of covariance at each landmark to produce a pattern that is not strictly symmetric as in the third test of the first experiment, but which varies in a more complex, and arguably more “biological” manner than any of the examples in the first experiment.

The first test of Experiment 4 used parameters identical to the first in Experiment 2 as a reference (Fig. 4D**;** CR = 1.27; *P* = 0.96), but in the second two tests (Fig. 4E, F**)** the directions of variation at each landmark were randomly rotated by 0° to 360°. In both cases, the effect was to dramatically constrain the position of the centroid with respect to the variation in the landmarks, to improve recoverability as measured by CR (CR = 0.42 and 0.74; *P* < 0.001 and 0.01), and to recover the original modular patterns accurately. While Procrustes superimposition had a small effect on the covariance matrix and the perceived closeness of relation between landmarks in each module, this effect was minimal.

The results of the first experiment can now be reinterpreted in light of the fourth: it is not symmetric shape variation that matters as much as the lack of systematically directional variation. In both the first and second tests of the first experiment, the direction of variation at all landmarks was somewhat parallel. In the first experiment all of the landmarks shared half of their variation as a vertical component, whereas in the second experiment all of the landmarks that varied shared their direction. The symmetrical pattern in the third test of the first experiment performed no better than the random patterns in the second and third tests of the fourth experiment. Regardless of whether the landmark variation is directionally random or symmetrical, the effect is to severely constrain variation in the position of the centroid relative to the landmarks, and therefore to minimize the effects of Procrustes superimposition on the covariance matrix.

### Experiment 5: Direction of covariance and number of landmarks

If the complexity of the directional variation matters, then more landmarks should increase that complexity if their direction of variation is independent. We tested that possibility in our fifth and final experiment (Fig. 4G–I). We used the same 8, 16, and 24 landmarks as in the third experiment, but this time randomly rotated the direction of variation at each landmark. When the major axis of variation at each landmark is oriented in a different direction, increasing the number of landmarks has a positive effect on the recoverability of modules. As the number of landmarks increased, the CR ratio declined (CR = 0.34 and *P* < 0.001 for *k *=* *8, CR = 0.17 and *P* = 0.00 for *k *=* *16 and CR = 0.18 and *P* < 0.001 for *k* = 24). With 24 landmarks with randomly varying directionality, Procrustes superimposition had little visible effect on the covariance pattern or on the modularity dendrogram (Fig. 4I).

### Further considerations on centroids and natural superimpositions

The original simulated shapes before Procrustes superimposition can be considered to be in their “natural” superimposition, especially if the base shape has a centroid size of one. The concept of “natural superimposition” warrants philosophical consideration. It is a biologically vague idea, yet the crux of the issue of whether Procrustes superimposition alters the “real” covariances between landmarks depends upon the idea of a “natural superimposition.” The strategy of the Procrustean paradigm in geometric morphometrics is to remove the so-called “nuisance” parameters of size, translation, and rotation by translating landmarks (and semilandmarks) so that the centroid of each shape is at the origin, scaling them to have centroid size of one, and rotating them to minimize the sum-of-squared distance between shapes. Upon completion of the superimposition, the new shape data are placed in a single comparable coordinate system where their differences can be analysed, analogous to mean-centering normal variables and standardizing them to unit variance. The strategy we adopt here assumes that individuals are generated by some process (e.g., ontogenetic development) that produces variants on a general theme (our base landmark configuration, which we refer to as the *archetype* after Richard Owen’s notion that vertebrate species were all variations on an underlying theme) with a covariance structure **V** that arises from the generating process. Since our modeling procedure (Fig. 3A) generates residual variation from a multivariate normal covariance distribution with a mean of zero, the shapes are invariant with respect to translation and rotation; and since the residuals are all added to the same *archetypal* configuration of landmarks (and semilandmarks), they are also invariant in scale with respect to the process that generated them.

Individual simulated shapes, however, do not have a centroid size of one, their individual centroids are not aligned, they are not in optimal alignment, and their shapes are not the same as the *archetype*. Figure 3C shows two simulated shapes along with their centroids to illustrate this fact. Instead, having a centroid size of one, a centroid centred at the origin, and an archetypal shape are properties of the mean of the simulated shapes (Fig. 3D). Thus, the simulated shapes are not aligned using Procrustes superimposition, but they are in the optimal alignment with regard to the process that generated them. This difference between the two alignments is the source of Procrustes-induced covariance patterns. Accurately representing the natural superimposition, and thus the processes generating shape variation, is a critical concern in most analyses employing geometric morphometrics, and thus understanding the cause of these deviations is an important theoretical and practical consideration.

The reason why the centroids are not perfectly aligned is because the generating process used in these examples makes no explicit reference to the centroid. Instead, the generating process produces random deviations from an archetypal configuration of landmarks with a modular covariance pattern. Each deviation has its own centroid, centroid size, and orientation relative to the archetype. One can imagine other generating processes that do make reference to the centroid (or, at very least, to a landmark that has an invariant position). For example, the development of the tribosphenic molar involves a process of tissue growth that begins with the apex of a particular tooth cusp (the protoconid) and via a cascade of molecular signaling and folding produces additional cusps in a complex pattern around the original one (Jernvall 1995; Thesleff and Sahlberg 1996). One can therefore say that the natural alignment of tribosphenic tooth shapes is invariant at the protoconid cusp tip with a variance and covariance structure determined by the cascade of subsequent cusp formation. Polly (2005) simulated tooth shapes using an analogous cascading process that started with the protoconid landmark. But even in this example, the protoconid cusp is not equivalent to the centroid, which varies in its relative position depending on the arrangement of other cusp landmarks.

If there were a generating process that began with an object’s centroid, such as development of a radially symmetric structure like a coral polyp (cf., Budd et al. 1994) the “natural” and Procrustes superimpositions could be nearly identical once standardized for size, rotation, and translation. But, as our experiments show, a complex pattern in the direction of variation around landmarks with respect to one another coupled with strong covariance has the effect of constraining the location of the centroid, regardless of the generating process. The greater the complexity, the greater the constraint on the centroid position, and the more similar the “natural” and Procrustes superimpositions.

Presuming that real biological shapes have similar directional diversity of landmark variation within modules as in our fifth experiment, our results suggest that Procrustes superimposition is unlikely to interfere with the recoverability of modular patterns, even when the number of landmarks is small. Properties that matter for recoverability of modular patterns include: (1) variation in directional variation within and between modules and (2) centroids whose “natural” position varies little in proportion to variation in individual landmarks. Properties that do not matter for recoverability of modular patterns include: (1) total number of landmarks (or semilandmarks) and (2) absolute magnitude of shape variation.

Thus, on the question of whether the use of sliding semilandmarks exacerbates the effect of Procrustes superimposition on covariance structure (Cardini 2019), the results of our third experiment suggest that adding landmarks neither improves nor inhibits the recoverability of modules. The fact that the direction of variation in sliding semilandmarks tends to be fairly uniform as a result of their fitting procedure (e.g., Perez et al. 2006) suggests that they will not improve recoverability to the same extent as covarying landmarks (or non-sliding semilandmarks) whose direction varies with respect to one another. However, sliding semilandmarks improve representation of complex structures, such as surfaces, far beyond the abilities of landmarks, and thus the increased complexity, and added variation in directionality of variation, will constrain centroid variation, improve the Procrustes fit relative to the “natural superimposition,” and thus increase the accuracy of recovering modules for biological structures.

### Comparing analyses of integration with landmark and semilandmark datasets

In the above sections, we demonstrate that high-density semilandmark datasets add important detail on morphology beyond that which is captured by Type I/II landmarks. In addition, our simulations indicate that Procrustes superimposition does not mislead analyses of integration in biologically realistic scenarios, that is, those with complex directions of variation sampled by geometric morphometric data, regardless of number of landmarks or semilandmarks. Finally, we address the question of how using semilandmarks in analyses of integration and modularity may change results and interpretations of these quantities, compared to analyses based on landmarks alone. Because semilandmarks and sliding semilandmarks are not independent of each other due to their fitting procedure, there are expected effects on analyses of integration and modularity. Specifically, adjacent semilandmarks and sliding semilandmarks will be correlated because their placement is relative to each other, in addition to any biological correlation amongst the structures they represent. The effect of this fitting may be to exaggerate the correlations or covariance of proximal semilandmarks relative to those farther away, which may increase the appearance of modularity across regions. On the other hand, landmarks (and also curves based on element boundaries) may have the opposite effect. Because Type I landmarks in a structure such as a skull will be largely limited to sutures between elements, they may suffer from boundary bias, exaggerating the apparent integration of those elements compared to aspects of their respective morphologies that are not located at their point of juncture. It is important to recognize that both approaches suffer from statistical artefacts due to the nature of the data collection approach and may have opposing biases in reconstructing trait integration and modularity. Thus, the comparison of results generated by these different approaches is critical for identifying the magnitude and impact of their respective biases and artifacts.

In two recent studies of variational or static (Marshall et al. 2019) and evolutionary (Bardua et al. 2019b) integration and modularity in caecilian crania, we conducted extensive analyses of integration across 16–17 cranial regions using 66 (*Idiocranium russeli*), 68 (*Boulengerula boulengeri*), or 53 (32 caecilian genera) landmarks and 1363-1558 curve and surface sliding semilandmarks. These datasets were analysed using CR analysis (Adams 2016) and a maximum likelihood approach (Goswami and Finarelli 2016), with allometric and phylogenetic (for the intergeneric analysis) corrections. In both studies, results were compared across analyses of the full dataset and analyses of the landmark-only datasets. In the intergeneric study of evolutionary modularity, both datasets significantly supported a highly modular pattern (16 module model, full dataset CR = 0.59, *P* < 0.01; landmark-only dataset CR = 0.88, *P* < 0.01). Despite supporting a modular pattern, the landmark-only dataset returned a CR much closer to one, indicating relatively more integration among modules. In particular, the major differences were increased integration of the bones forming the cranial vault, which, in landmark-only analyses are defined entirely by their sutures (mainly with each other), and reduced within-region integration in the landmark-only analyses, as expected (Fig. S2 in Bardua et al. 2019b). A similar result is observed in the intraspecific study of two species of caecilieans (Marshall et al. 2019), with all analyses again significantly supporting a highly modular skull. For example, CR analyses of the 17-module model for *I. russeli* were highly significant for the full dataset before (CR = 0.621, *P* < 0.001) and after (CR = 0.519, *P* < 0.001) allometric correction and with the landmark-only dataset before (CR = 0.851, *P* < 0.001) and after allometric correction (CR = 0.738, *P* < 0.001). As before, the landmark-only analyses returned CR values closer to one, suggesting more integration than the analysis of the full dataset, and removing allometric effects resulted in reduced CR values, supporting a more modular pattern. Despite this overall consistency across datasets and analyses, examination of the pairwise CR values between regions, in addition to the mean CR across the full cranium, suggests the allometry may have a stronger influence on landmark-only analyses. For example, in the *I. russeli* dataset, landmark-only analyses identify 49 out of 120 region pairs with CR values >0.9, with some exceeding a value of one (indicating integration). Following removal of allometry, only 16 region pairs show CR values >0.9, and the overall pattern of integration across regions is congruent with the analysis of the full dataset. Allometric correction did not have a similar effect on the analyses of the full dataset. These results, while supporting that analyses are largely consistent across datasets, suggest that allometry may have a stronger influence on recovered patterns of integration in landmark-only datasets. If so, this effect may reflect the tendency for many landmarks to be placed at element boundaries, resulting in a stronger signal of structure size relative to the complexity of its shape, with the latter being better captured by semilandmarks.

## Conclusions

Capturing and quantifying morphology using high-resolution imaging has opened the door to high-density morphometric data analysis with semilandmarks or pseudolandmarks. Our analyses on both simulated and empirical datasets demonstrate that semilandmarks provide far more comprehensive, as well as accurate, characterizations of morphological variation than analysis of landmarks alone, which suffer from limitations to points that can be identified repeatedly on specimens and often leave large areas of complex structures entirely unsampled. However, these gains in quantifying morphology raise questions about the biases that these datasets may bring, in terms of quality of data, procedural artefacts, and ability to accurately recover attributes such as trait integration. Here we demonstrate that some of the concerns with geometric morphometric analysis of trait integration and modularity are unlikely to affect analyses of complex structures, such as those encountered in biological specimens. We also demonstrate that increasing landmark or semilandmark sampling alone does not exacerbate issues with procedures such as Procrustes analysis. We further suggest that analyses incorporating semilandmarks may be less influenced by boundary bias and allometric effects, which may exaggerate degree of integration across regions in landmark-only analyses, while analyses of sliding semilandmark may exaggerate within- region integration and between-region modularity. It remains a continuing challenge to develop methods that alleviate these effects. In doing so, we should prioritize improving the representation of morphology, rather than limiting future studies to existing methods that quantify complex structures with a small number of lengths or landmarks and leave much of the available biological information unused (Collyer et al. 2015). Similarly, most existing methods for the analysis of phenotypic integration and modularity are overly simplistic and incapable of accurately conveying the complex hierarchy of relationships across traits. Furthermore, most of these methods have not been developed or tested for high-density datasets, which will certainly present new challenges as these datasets become increasingly common in studies of phenotypic integration and morphological evolution. It is thus critical to remember that all methods have costs and benefits, including both landmarks and semilandmarks. Nonetheless, the benefits of high-density geometric morphometrics for more precisely representing morphology solves many issues with reconstructing the evolution of complex structures across disparate taxa and is a promising path forward for “Big Data” approaches to comparative morphology.

## Notes

From the symposium “Comparative Evolutionary Morphology and Biomechanics in the Era of Big Data” presented at the annual meeting of the Society for Integrative and Comparative Biology, January 3–7, 2019 at Tampa, Florida.
